# Investigation of the Microstructural Evolution and Mechanical Properties of the AlCoCrFeNi_2.1_ EHEA Fabricated by Additive Manufacturing Assisted by Heat Treatment

**DOI:** 10.3390/ma18102330

**Published:** 2025-05-16

**Authors:** Xin Zhang, Wenxin Feng, Fanghui Jia, Wanhui Liu, Jian Wang, Lisong Zhu, Yangchuan Cai

**Affiliations:** 1 School of Automobile Engineering, Suzhou University of Technology, Changshu 215500, China; liuwh@cslg.edu.cn (W.L.); jianwang@cslg.edu.cn (J.W.); 2 School of Materials Science and Engineering, Tianjin University of Technology, Tianjin 300384, China; fengwenxin01@163.com (W.F.); 18622265848@163.com (Y.C.); 3 Baosteel Research Institute, Shanghai 201900, China; 4 School of Mechanical, Materials, Mechatronic and Biomedical Engineering, University of Wollongong, Wollongong, NSW 2522, Australia; lz131@uowmail.edu.au; 5 Ningbo Qiyi Metal Co., Ltd., Ningbo 315622, China

**Keywords:** AlFeCoCrNi_2.1_ EHEA, laser additive manufacturing, heat treatment, recrystallization

## Abstract

Eutectic high-entropy alloys (EHEAs) exhibit excellent casting properties and comprehensive mechanical performance, making them suitable for fabricating spatial engineering components using additive manufacturing techniques. However, the rapid solidification process also leads to increased internal stress and reduced structural stability in the components. Therefore, this study focuses on the AlFeCoCrNi_2.1_ EHEA as the research subject, utilizing laser additive manufacturing to fabricate components and systematically investigating the influence of heat treatment processes on the microstructure and mechanical properties of the components. The research demonstrates that low-temperature heat treatment (700 °C and below) acts as a stress relief-annealing process for the components. The yield strength decreased from 1003.2 MPa to 742.1 MPa. At 900 °C heat treatment, the constraining effect between recrystallized grains and surrounding grains outweighs the dislocation release effect caused by recrystallization, resulting in an increase in dislocation density. The yield strength remained approximately stable at around 730 MPa. High-temperature heat treatment (1100 °C) alters the orientation of phase structures and fragments the two-phase structure through recrystallization, leading to generally stable mechanical properties of the components. The yield strength of the cast components further decreased to 582.6 MPa, while that of the LMD-fabricated parts retained stability at approximately 730 MPa.

## 1. Introduction

Eutectic high-entropy alloys (EHEAs) have emerged as a focus in high-performance materials research due to their exceptional mechanical properties and high-temperature stability [1,2,3]. In recent years, researchers have fabricated EHEAs through various manufacturing processes, such as casting, selective laser melting (SLM), and direct energy deposition (DED), and they further investigated the regulatory effects of heat treatment on their microstructure and mechanical properties. By synthesizing recent research advances, this review specifically addresses the critical role of heat treatment in optimizing the performance of EHEAs.

Heat treatment has been demonstrated to facilitate the microstructural optimization of as-casting AlCoCrFeNi_2.1_ EHEAs [4,5,6]. Researchers, including Sudeep Kumar T. et al., have found that homogenization annealing effectively reduces compositional segregation in the as-casting microstructure of AlCoCrFeNi_2_, thereby enhancing the alloy’s uniformity [5]. In dual-phase EHEAs, heat treatment can regulate phase proportions and phase boundary structures, leading to improved mechanical properties [7]. Consequently, appropriate heat treatment not only enhances the strength and ductility of the alloy but also ensures excellent mechanical stability across a range of temperature environments [4,6].

Laser additive manufacturing (LAM) offers unparalleled advantages in the efficient and high-precision fabrication of components with complex geometries, surpassing traditional manufacturing methods [8]. The exceptional casting ability of EHEAs enables their effective integration with AM techniques, facilitating the production of high-quality, high-performance structural parts [9,10]. However, the AM process involves rapid solidification, resulting in non-equilibrium atomic arrangements and interfacial configurations. Therefore, post-process heat treatment is essential to optimize the microstructure and enhance the mechanical properties of the fabricated components. Y.W. Yu [11] and H.L. Gao [12] fabricated Ni_30_Co_30_Cr_10_Fe_10_Al_18_W_2_ EHEAs using SLM, and the alloy exhibited an excellent strength–ductility balance after heat treatment. Similarly, Z.H. Fu et al. [13] observed that the lamellar structure of SLM-prepared AlFeCoCrNi_2.1_ EHEAs was retained post-heat treatment, significantly influencing the material’s strength and ductility. However, different heat treatment conditions can lead to markedly distinct mechanical properties. For instance, annealing may promote grain growth, precipitate evolution, and internal stress reduction, but it can also result in a decrease in yield strength [14]. L.W. Lan [15] and P. Peng [16] produced AlFeCoCrNi_2.1_ EHEAs via DED, and heat treatment similarly induced significant microstructural transformations, such as phase structure changes and phase boundary strengthening. Consequently, the deformation mechanisms of heat-treated EHEA components during large plastic deformations, such as grain boundary sliding and dislocation strengthening, may be significantly influenced [7,17].

In summary, heat treatment-assisted AM has proven effective in regulating the structural stability (e.g., residual stress relief) and comprehensive mechanical properties of EHEA components, making them more suitable for high-performance structural applications. The wide variety of additive manufacturing technologies and types of EHEAs leads to significant differences in both the microstructure and macrostructure of fabricated alloy components. Furthermore, the selection of heat treatment processes critically influences these structural characteristics at both microscopic and macroscopic scales. Consequently, establishing clear application-driven relationships among additive manufacturing technologies, alloy types, and heat treatment parameters presents a complex challenge in this field. Addressing this challenge will significantly advance the engineering application and widespread adoption of EHEA-based additive components. However, resolving this issue requires concerted efforts from researchers to systematically develop and refine comprehensive models that correlate additive manufacturing methods, alloy compositions, and heat treatment protocols.

Laser metal deposition (LMD) is a type of laser additive manufacturing technology. When integrated with robotic arms, it enables the fabrication of complex components, and unlike SLM technology, it is not constrained by the volume limitations of the working chamber [18,19,20]. Therefore, in this study, LMD was employed to fabricate AlFeCoCrNi_2.1_ EHEA components, with as-casting AlFeCoCrNi_2.1_ samples serving as reference materials. The systematic investigation of the effects of heat treatment on microstructures aims to enrich the understanding of the heat treatment–microstructure–mechanical property relationships in the field of EHEA components fabricated via AM.

## 2. Experimental Procedures

### 2.1. Standard and Process for Establishment of EHEAs

The substrate used for additive manufacturing in this study was 316 stainless steel, which was machined and polished to dimensions of 300 mm × 300 mm × 300 mm. AlFeCoCrNi_2.1_ EHEA powders (Jiangsu Vilory Advanced Materials Technology Co., Ltd., Xuzhou, China), sourced commercially, were utilized for LMD. The powders had a molar ratio of alloying elements of approximately 1:1:1:1:2.1, with a purity level ranging from 99.0% to 99.5%. The powder particles were spherical, which was beneficial for fluidity during the powder-feeding process. The particle size distribution of the AlFeCoCrNi_2.1_ EHEA powder ranged from 45 to 150 µm, with an average particle size of 122 µm. The LMD process was performed using an HANSGS-RJ0016-F3K laser machine (Han’s Laser Technology Industry Group Co., Ltd., Shenzhen, China) under the following parameters: laser power of 1300 W, scanning speed of 6 mm/s, laser beam spot diameter (negative defocus) of 3 mm, powder feeding rate of 3.0 rpm (approximately 30 g/min), argon shielding gas flow rate of 25 mL/min, and a multi-track overlap ratio of 30%. The final fabricated LMD component measured 50 mm in length, 50 mm in width, and 15 mm in height.

For comparative purposes, AlFeCoCrNi_2.1_ EHEA casting samples were also prepared using a conventional casting method. The casting process was carried out in a KUSITE K-ZGF-0.04 suspension melting furnace (KUSITE, Zhengzhou, China). The raw materials included aluminum (99.99%), iron (99.9%), cobalt (99.98%), chromium (99.98%), and nickel (99.99%) powders, with a particle size range of 50–100 μm. High-purity argon gas (99.999%) was used as a protective atmosphere during melting. To ensure compositional homogeneity, the melting process was repeated four times. The final dimensions of the cast component were 50 mm in diameter and 50 mm in height.

Both cast and additively manufactured samples of the AlFeCoCrNi_2.1_ EHEA were heat-treated in a box furnace (Nabertherm GmBH, Lilienthal, Germany). The samples were heated to target temperatures (500 °C, 700 °C, 900 °C, 1100 °C) at a rate of 10 °C/min, held at these temperatures for 6 h, and subsequently furnace-cooled to room temperature.

### 2.2. Microstructure Characterization

Wire cutting was used to prepare samples with dimensions of 10 mm × 10 mm × 5 mm for phase structure and microstructure characterization. The samples were sequentially ground using 80-, 120-, 240-, 600-, 800-, 1000-, 1200-, and 1500-grit sandpaper, followed by polishing with a 0.25 μm particle size polishing compound on a polishing cloth until the surfaces were rendered scratch-free. Metallographic samples were etched using nitrohydrochloric acid (a mixture of nitric acid and hydrochloric acid in a 1:3 ratio) for approximately 20 s. The microstructures of the AlFeCoCrNi_2.1_ EHEAs, both in their as-prepared and heat-treated states, were examined using a Hitachi S-3400N scanning electron microscope (SEM; Hitachi High-Tech, Tokyo, Japan). Electron backscattered diffraction (EBSD) analysis was performed on a TESCAN MAIA3 system (TESCAN Group, Bron, Czech Republic) equipped with Aztec software (version Aztec 6.1, Oxford Instruments, Oxfordshire, UK) and Nordlys II(S) high resolution EBSD camera (Oxford Instruments, Oxfordshire, UK). To reduce strain artifacts introduced during sample preparation, the specimens were subjected to ion beam etching at a voltage of 6.5 kV for 30 min. For EBSD analysis, step sizes of 0.2 μm and 0.1 μm were selected to ensure high-resolution mapping. This rigorous methodology was designed to achieve precise and reliable microstructural characterization.

### 2.3. Mechanical Properties

Wire cutting was used to prepare cylindrical samples with a diameter of 4 mm and a height of 6 mm for compression testing. Compression tests were conducted using a CSS-44100 electronic universal testing machine (CCTM, Changchun, China) at a constant loading rate of 1 mm/min. For each material condition, two compression test specimens were prepared, and the final mechanical data were derived through fitting procedures. The compression test followed the ASTM E9 standard [21]. The fracture surfaces of the tested samples were examined using the SEM to analyze the failure mechanisms and gain insights into the material’s behavior under mechanical stress.

## 3. Results and Discussion

**Figure 1:**
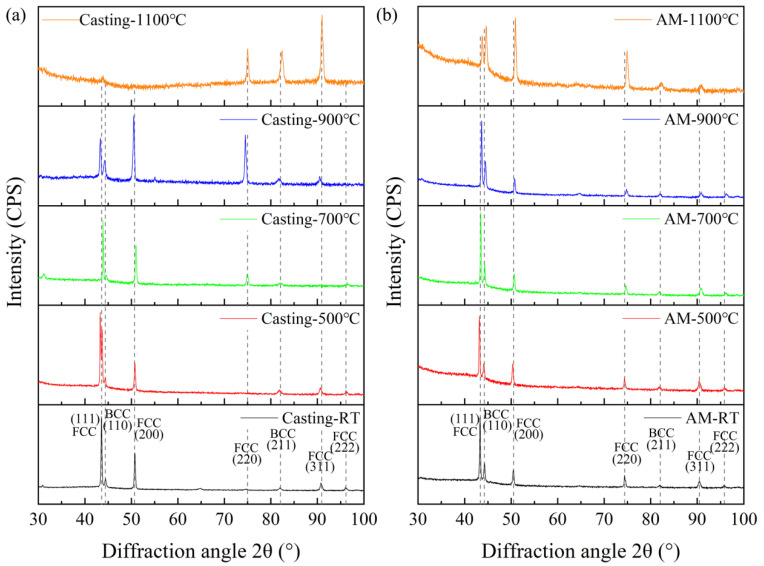
XRD results of the AlFeCoCrNi_2.1_ EHEAs before and after heat treatment. (**a**) Casting samples; (**b**) additively manufactured samples.

Prior to heat treatment, both the casting and additively manufactured (AM) samples exhibited a dual-phase structure consisting of FCC and BCC phases. The FCC phase was predominantly characterized by the (111) plane, with a minor contribution from the (200) plane, while the BCC phase was primarily dominated by the (110) plane, as illustrated in Figure 1. For the casting samples, the crystallographic composition of both phases remained unchanged up to a heat treatment temperature of 900 °C. At 900 °C, the FCC phase transitioned to a dominant (200) plane, with secondary contributions from the (220) and (111) planes, while the BCC phase retained its primary (110) orientation. When the heat treatment temperature was increased to 1100 °C, the FCC phase was primarily characterized by the (311) plane, with a minor contribution from the (220) plane, and the BCC phase completely transformed to the (211) plane. In contrast, for the AM samples, the FCC phase maintained its dominant (111) orientation, with a minor (200) contribution, and the BCC phase remained primarily (110)-oriented up to a heat treatment temperature of 1100 °C. At 1100 °C, the FCC phase shifted to a dominant (200) plane, with secondary contributions from the (111) and (220) planes, while the BCC phase retained its primary (110) orientation. Based on the XRD results, it is evident that the phase structure of the casting samples underwent significant changes under heat treatment. In contrast, the AM samples demonstrated remarkable phase stability at lower heat treatment temperatures (≤900 °C), although higher temperatures still induced phase transformations.

**Figure 2:**
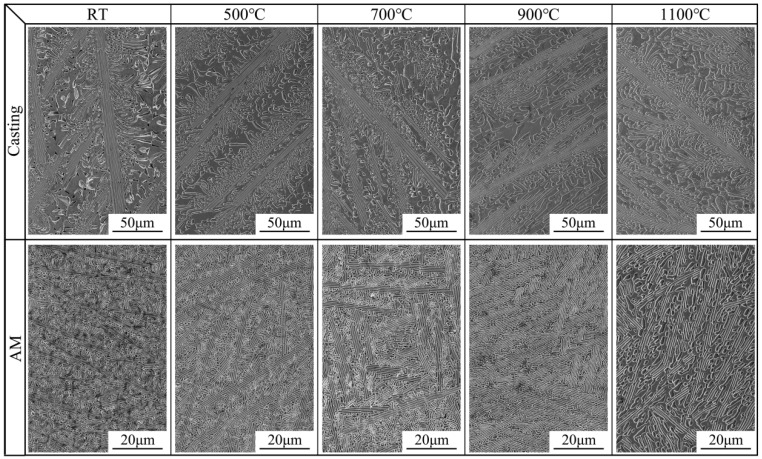
Microstructures of the AlFeCoCrNi_2.1_ EHEAs before and after heat treatment.

The SEM morphology of the casting and LMD samples revealed a distinct lamellar eutectic structure. At the final stages of solidification, changes in solute concentration and a reduced temperature gradient led to the transformation of the eutectic morphology into irregular shapes [22]. Additionally, the lamellar eutectic and irregular eutectic spacing in the AM samples was significantly finer than that in the casting samples, as shown in Figure 2 and Table 1. On one hand, the rapid cooling during the AM process generates high thermal gradients; on the other hand, the substrate or underlying additive-deposited layers act as nucleation substrates to promote heterogeneous nucleation. This dual effect significantly enhances the nucleation rate within the AM melt pool, thereby achieving grain refinement [23]. With increasing heat treatment temperatures, the casting samples maintained the integrity of their eutectic morphology, with no significant changes in grain or phase structure size. Similarly, the AM samples demonstrated excellent stability in eutectic morphology, grain size, and phase structure dimensions after heat treatment at temperatures below 900 °C. However, after heat treatment at 1100 °C, the lamellar eutectic structure in the AM samples was not evident, and a noticeable increase in phase structure dimensions was observed, as shown in Table 1.

**Figure 3:**
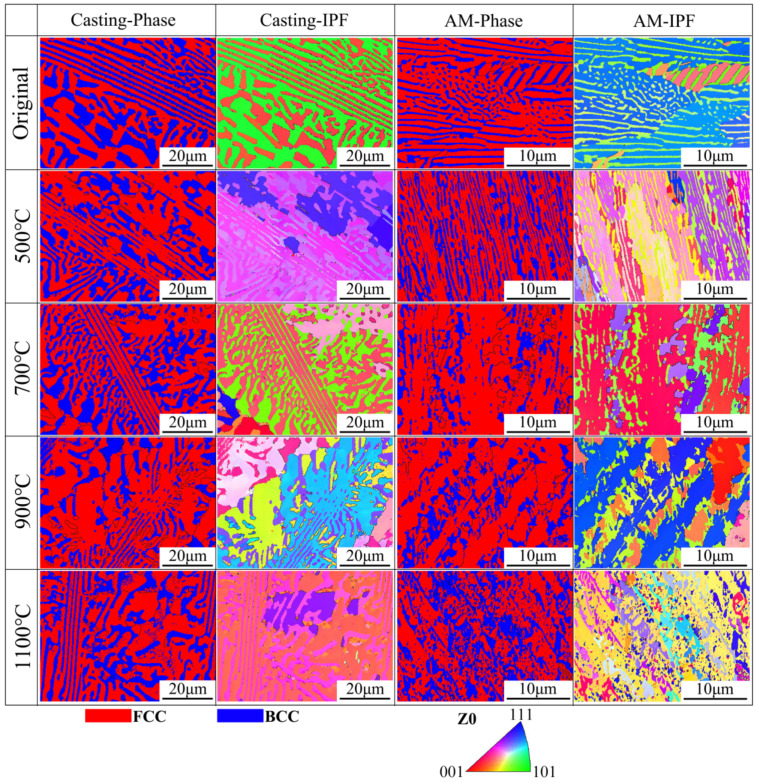
EBSD phase and IPF maps of the AlFeCoCrNi_2.1_ EHEAs before and after heat treatment.

To further investigate the evolution of eutectic morphology and phase structure in both casting and AM samples under heat treatment, EBSD was employed to analyze the phase distribution (with red representing FCC and blue representing BCC) and inverse pole figure (IPF), as shown in Figure 3. First, both the casting and AM samples consisted of FCC and BCC phases, consistent with the XRD results. Second, the casting samples exhibited excellent stability in eutectic morphology, with no significant changes observed, whereas the AM samples showed fragmented eutectic morphology and a loss of lamellar characteristics after heat treatment at 1100 °C. Finally, the IPF analysis revealed that the phase orientation within individual grains of the casting samples was highly uniform, while adjacent grains exhibited distinct IPF variations. A similar phenomenon was observed in the AM samples under low-temperature heat treatment (below 1100 °C). However, after heat treatment at 1100 °C, the uniformity of phase orientation within grains underwent a qualitative transformation. Combined with the preceding experimental results, it is evident that 1100 °C represents a critical threshold for heat treatment in both casting and AM samples, significantly influencing their microstructural evolution.

**Figure 4:**
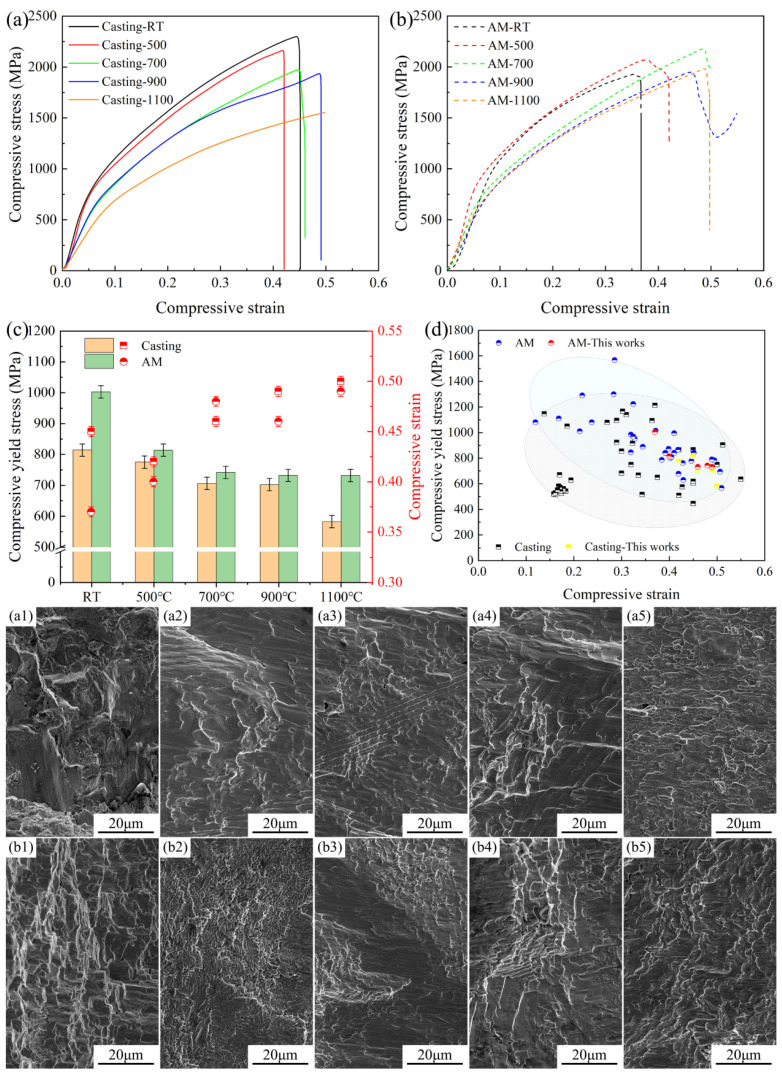
Experimental results of mechanical properties of AlFeCoCrNi_2.1_ EHEAs before and after heat treatment. (**a**) Compression curves of casting samples; (**b**) compression curves of AM samples; (**c**) compression test data; (**d**) summary of the literature data; (**a1**–**a5**) compression fracture microstructures of casting samples; (**b1**–**b5**) compression fracture microstructures of AM samples.

In this study, the compressive properties of both casting and AM samples subjected to heat treatment were systematically evaluated and analyzed, as illustrated in Figure 4. The stress–strain curves obtained from the compression tests are shown in Figure 4a,b, with compressive strain and compressive yield strength serving as the primary parameters for analysis. Regardless of heat treatment, the AM samples consistently exhibited higher compressive yield strength but lower compressive strain compared to the casting samples, as depicted in Figure 4c. With increasing heat treatment temperatures, the compressive yield strength of the casting samples gradually decreased, while the compressive strain showed a corresponding increase. Similarly, the compressive strain of the AM samples also increased with higher heat treatment temperatures; however, the compressive yield strength exhibited only a marginal decrease after 700 °C. This suggests that the AM samples possess superior resistance to high-temperature softening compared to the casting samples. By summarizing the compressive properties of EHEAs with FCC+BCC phase structures, it is evident that despite changes in eutectic morphology and phase structure induced by heat treatment, the mechanical properties of both casting and AM samples remained within an optimal range, as shown in Figure 4d.

Furthermore, the fracture surface morphology of the samples after compression testing revealed distinct patterns. For the casting samples heat-treated below 900 °C, the fracture surfaces exhibited clear eutectic features, and the shear plastic deformation of the FCC phase was immediately adjacent to the fracture surfaces, as shown in Figure 4(a1–a4). In this study, compression testing was employed. During the fracture process, the opposing fracture surfaces undergo mutual friction upon further compression, causing the plastically deformed FCC phase to adhere closely to the fracture surfaces. Nevertheless, this phenomenon does not alter the consistency of the overall fracture mechanism with that reported in Ref. [24], where the BCC phase preferentially undergoes brittle fracture to promote crack propagation, while the FCC phase exhibits ductile shear fracture characteristics. Although the AM samples exhibit finer phase dimensions, the compression fracture morphology of the samples subjected to the same heat treatment process closely resembles that of their cast counterparts, as illustrated in Figure 4(b1–b4). However, no obvious eutectic morphology was observed in the fracture surfaces of casting samples heat-treated at 1100 °C, even though SEM and EBSD analyses confirmed the presence of a eutectic structure, as illustrated in Figure 4(a4). This originates from the high plasticity of samples under this heat treatment condition, where the extensive plastic shear deformation within the FCC phase masks the brittle-fractured BCC phase. In contrast, for AM samples subjected to identical conditions, the lamellar eutectic structure is significantly disrupted, rendering the fracture surfaces devoid of eutectic features.

**Figure 5:**
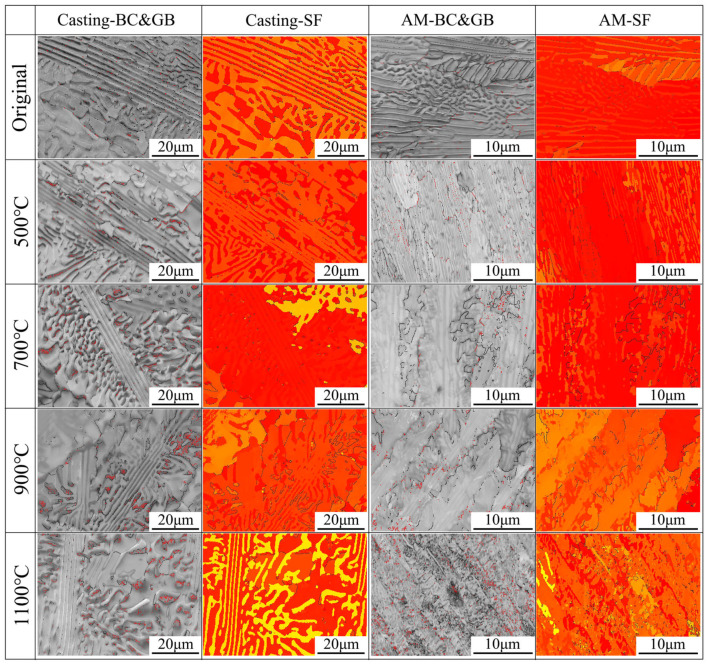
BC, GB, and SF maps of the AlFeCoCrNi_2.1_ EHEAs before and after heat treatment.

To systematically investigate the influence of heat treatment on the mechanical properties of casting and additively manufactured components, this study initially employs EBSD to characterize and analyze the differences between the two types of materials and their coupling relationships with heat treatment processes through BC (boundary character), GB (grain boundary), and SF (Schmid factor) representations, as illustrated in Figure 5. In the BC images, red lines represent low-angle grain boundaries (<2°), while black lines denote high-angle grain boundaries (>10°). The low-angle grain boundaries in both casting and additively manufactured parts are predominantly located at grain boundaries and phase boundaries. Overall, prior to heat treatment at 900 °C, the content of low-angle grain boundaries in both casting and additively manufactured parts slightly increases; after heat treatment at 1100 °C, the increase in low-angle grain boundary content becomes more pronounced, with a more uniform distribution observed in the additively manufactured parts. The SF reflects the relative deformability of different regions within a sample [25]. Theoretically, low-angle grain boundaries (approximating dislocations) exert a negative influence on the deformability of materials, meaning that regions with a higher content of low-angle grain boundaries tend to exhibit weaker deformability. However, the distribution pattern of SF within the sample does not align with that of low-angle grain boundaries, indicating the presence of more significant influencing factors. Notably, the distribution of SF shows a positive correlation with the IPF distribution of the sample. Under low-temperature heat treatment conditions (1100 °C), the distribution pattern of SF is primarily characterized by the following aspects: (1) when the orientation difference between adjacent grains is significant, the distribution follows a grain-based pattern; (2) when the orientation difference between adjacent grains is minor, the distribution follows a phase-structure-based pattern; (3) in additive manufactured parts, the directional growth of grains and phase structures results in relatively small differences in IPF and SF between phase structures and adjacent grains.

The distribution pattern of the SF in casting parts after heat treatment at 1100 °C adheres to the aforementioned second rule, but the differences become significantly more pronounced. Researcher T. Xiong [26] discovered through studies that the Kurdjumov–Sachs (K-S) relationship between the FCC and BCC phases in AlCoCrFeNi_2.1_ eutectic high-entropy alloys satisfies the following orientations for optimal coordinated deformation capability: (-1-11)_FCC_‖(101)_BCC_ + [1-10]_FCC_‖[1-1-1]_BCC_, (112)_FCC_‖(321)_BCC_ + [-110]_FCC_‖[-111]_BCC_, and (33-2)_FCC_‖(01-1)_BCC_ + [-110]_FCC_‖[-111]_BCC_. In casting parts after heat treatment at 1100 °C, the FCC phase structure exhibits a (311) plane, while the BCC phase structure exhibits a (211) plane, both of which are non-close-packed planes with relatively weak coordinated deformation capabilities. Consequently, significant differences in SF between the phase structures are observed. In contrast, for additive manufactured parts, the lamellar eutectic structure nearly disappears after heat treatment at 1100 °C, leading to the loss of the inherent coordinated deformation capability of the eutectic structure. Therefore, even if the FCC phase structure transitions to a non-close-packed (200) plane, the differences in SF between phase structures remain less pronounced compared to casting parts under the same conditions.

**Figure 6:**
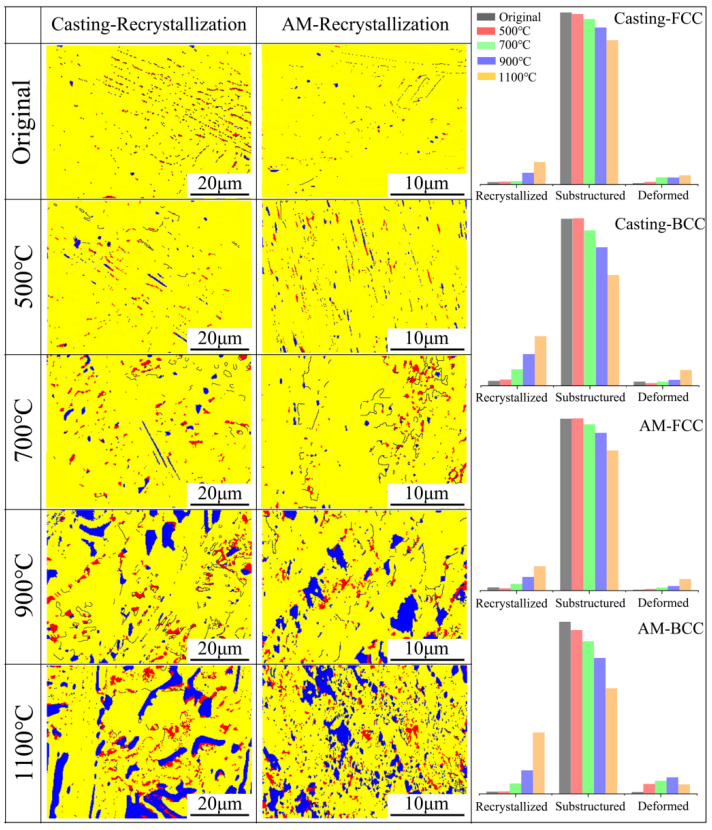
Recrystallization states of the AlFeCoCrNi_2.1_ EHEAs before and after heat treatment.

Figure 6 illustrates the recrystallization distribution in both types of materials before and after heat treatment, where blue represents recrystallized, red represents deformed, and yellow represents substructure. Prior to heat treatment at 700 °C, both FCC and BCC phases in the two types of samples are predominantly substructure. At 700 °C heat treatment, a small number of recrystallized regions begins to appear in the BCC phase of both samples, indicating that this heat treatment temperature promotes the formation of recrystallization, which aligns with other findings [27,28]. After heat treatment at 900 °C, a significant increase in recrystallized regions is observed in both FCC and BCC phases of the two samples, with the amount gradually increasing as the heat treatment temperature rises. Similarly to the 700 °C condition, the BCC phase in the samples exhibits a higher number of recrystallized grains, which is attributed to the higher structural stability of the FCC phase compared to the BCC phase at elevated temperatures [29]. Additionally, starting from the 700 °C heat treatment, deformed grains appear in both FCC and BCC phases, resulting from the formation of recrystallized grains and the constraints imposed by surrounding grains. Notably, at 1100 °C heat treatment, the deformation in the BCC phase of the casting sample is more pronounced than in the corresponding phase of the additively manufactured sample. This phenomenon is closely related to the transformation in the coordination relationship between the FCC and BCC phase interfaces, as detected by the XRD analysis mentioned earlier.

**Figure 7:**
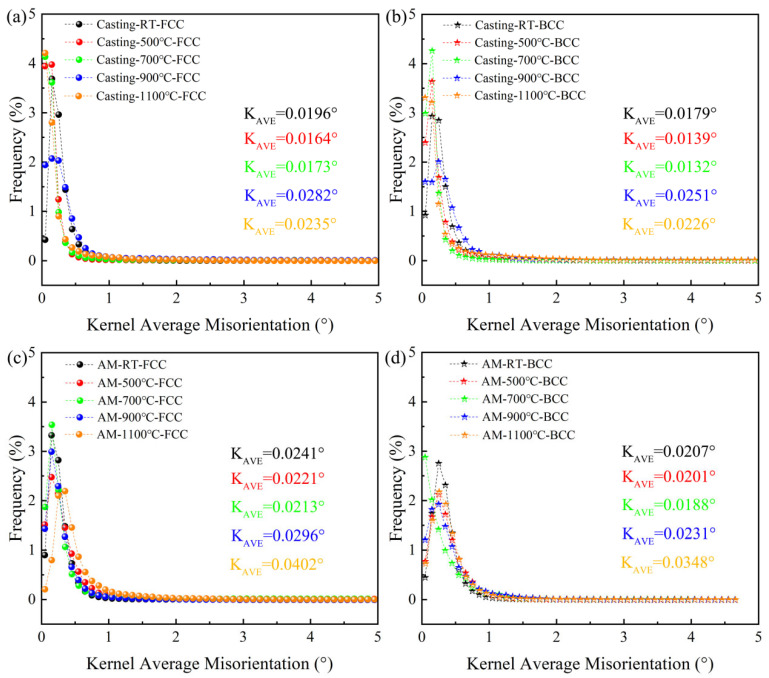
KAM value distributions of the AlFeCoCrNi_2.1_ EHEAs before and after heat treatment. (**a**) Casting FCC; (**b**) casting BCC; (**c**) AM FCC; (**d**) AM BCC.

**Figure 8:**
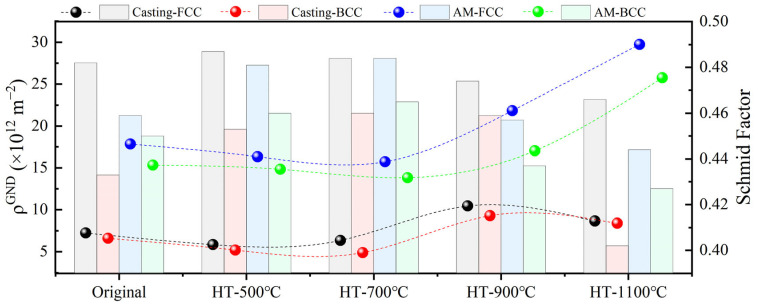
ρ^GND^ and SFs of the AlFeCoCrNi_2.1_ EHEAs before and after heat treatment.

The local misorientation was conducted to study the evolution and density of geometrically necessary dislocation (GND). Kernel average misorientation (KAM) was used to determine local dislocations from the obtained EBSD data [30]. The average value of KAM can be used to quantitatively calculate the GND density (ρ^GND^) using Equation (1) [31]:

(1) $$\rho^{GND}=\frac{2{KAM}_{ave}}{\mu b}$$

where μ is the step size (0.2 μm and 0.1 μm), b is the Burgers vector (2.35 × 10^−10^), and KAM_ave_ represents the KAM’s weighted average for the selected areas. Figure 7a–d illustrates the KAM_ave_ distribution curves for both casting and additively manufactured parts before and after heat treatment. The KAMave values for the FCC and BCC phases in both types of materials were calculated using the weighted average method and are detailed in Figure 7. By combining the KAM_ave_ values from Figure 7 and Equation (1), the ρ^GND^ was derived, as shown in Figure 8. Overall, the dislocation density in the additively manufactured parts is significantly higher than that in the casting parts. This is attributed to the non-equilibrium rapid solidification process inherent to additive manufacturing, which on one hand leads to insufficient atomic arrangement and subsequent atomic misalignment and on the other hand increases the constraints between the two-phase structures, grains, and adjacent melt pools, ultimately resulting in a higher dislocation density in the components. The dislocation density in the FCC phase is consistently higher than that in the BCC phase, which is associated with the FCC phase’s greater capacity to accommodate dislocations [29].

Before heat treatment at 700 °C, the dislocation density in both types of materials gradually decreases, which is attributed to dislocation release under heat treatment conditions and the onset of minor recrystallization at 700 °C. At 900 °C heat treatment, the dislocation density increases significantly due to enhanced recrystallization in the two-phase structures and the mutual constraints between the original two-phase matching relationships and the recrystallized phase structures. At 1100 °C heat treatment, the dislocation density in the casting parts decreases partially due to further increases in recrystallized grain content and the release of dislocations through continuous dynamic recrystallization. In contrast, the dislocation density in the additively manufactured parts increases further due to phase structure fragmentation, which intensifies the constraints between the two phases. Figure 8 also illustrates the relationship between the evolution of SF values and ρ^GND^ for the two phases in both materials. It is evident that the two datasets generally exhibit a negative correlation, except for the casting parts after 1100 °C heat treatment, where a positive correlation is observed. This anomaly can be partially explained by the phase structure orientation changes revealed in the XRD analysis (Figure 1) and SF analysis results (Figure 5).

**Figure 9:**
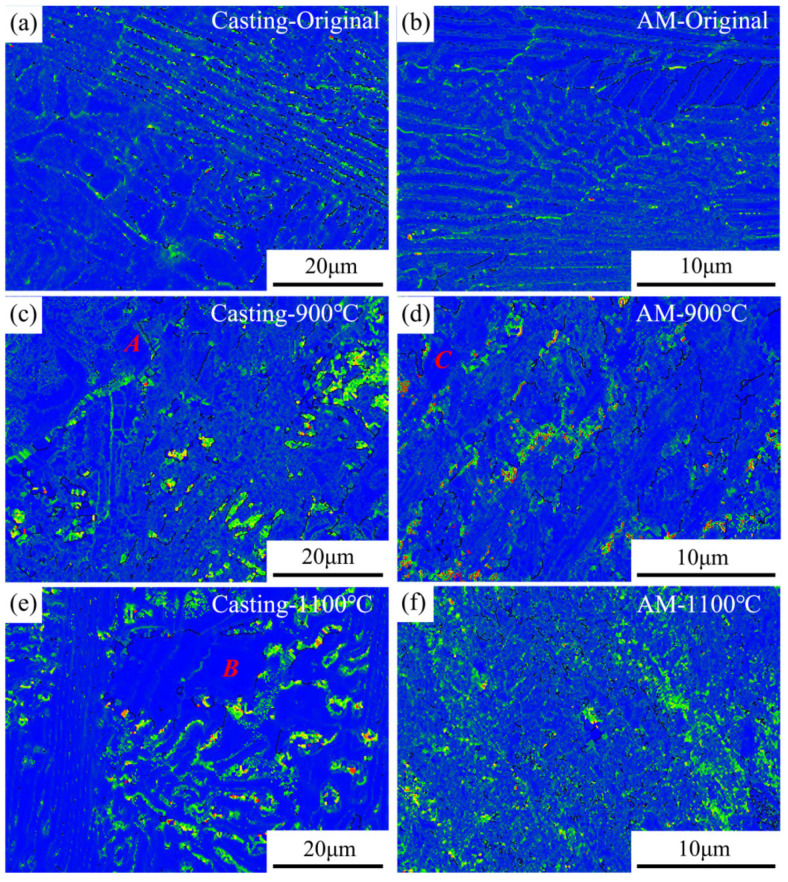
KAM map distributions of the AlFeCoCrNi_2.1_ EHEAs before and after heat treatment: (**a**) casting original; (**b**) AM original; (**c**) casting 900 °C; (**d**) AM 900 °C; (**e**) casting 1100 °C; (**f**) AM 1100 °C.

To further elucidate the changes in dislocation density in both materials after heat treatment at 900 °C, the distribution of KAM_ave_ is presented, as shown in Figure 9. After heat treatment at 900 °C, a significant amount of recrystallization occurs in both samples. On one hand, this leads to a notable reduction in dislocation density in certain recrystallized regions (e.g., regions A and C in Figure 9). On the other hand, the non-uniform distribution of recrystallized regions and surrounding original grains generates substantial constraints, resulting in an increase in dislocation density. After heat treatment at 1100 °C, the content of recrystallized grains in the casting parts further increases, and the dislocation density released by recrystallization outweighs the dislocation density generated by intergranular constraints (e.g., region B in Figure 9). In contrast, the phase structure in the additively manufactured parts undergoes significant fragmentation, and the mutual constraints between grains are further enhanced, leading to a continuous increase in dislocation density.

**Figure 10:**
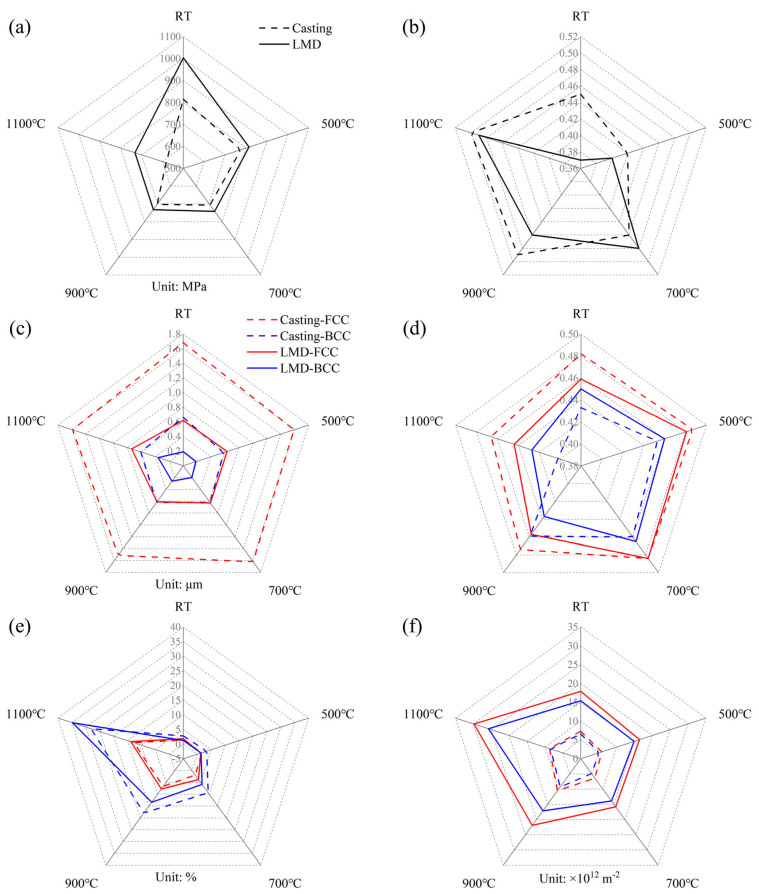
Strengthening mechanisms of AlFeCoCrNi_2.1_ EHEAs before and after heat treatment: (**a**) compressive yield strength; (**b**) compressive strain; (**c**) lamellar eutectic dimension; (**d**) Schmid factor (SF); (**e**) recrystallization state; (**f**) ρ^GND^.

In summary, the strengthening mechanisms of casting and additively manufactured parts before and after heat treatment are primarily reflected in the following aspects (Figure 10):

(1) The rapid solidification characteristics induced by laser additive manufacturing technology lead to a high nucleation rate within the melt pool, resulting in the refinement of lamellar eutectic structures and increased complexity in their growth direction, as shown in Figure 10c. Consequently, the mechanical properties of additively manufactured parts surpass those of casting parts, as shown in Figure 10a,b.
(2) Under heat treatment conditions at 700 °C and below, the heat treatment serves as a stress relief-annealing process, reducing the dislocation density in both materials, as shown in Figure 10f. Meanwhile, the phase structures and grain sizes of the two materials remain largely unchanged. Thus, the mechanical properties are primarily determined by the dislocation density.
(3) After heat treatment at 900 °C, the content of recrystallized grains in both materials increases. The mutual constraints caused by the non-uniform spatial distribution between these recrystallized grains and the surrounding grains lead to an increase in dislocation density, as shown in Figure 10e,f. However, the phase structures and grain sizes of the two materials remain largely unchanged. As a result, the mechanical properties are determined by the dislocation density and the presence of large-area recrystallized regions.
(4) After heat treatment at 1100 °C, the grain morphology of the casting parts shows little change, but the increased recrystallization content results in a further reduction in dislocation density. Additionally, the orientation of the two-phase structure undergoes a shift, as shown in Figure 10e,f. Therefore, the mechanical properties of the casting parts are determined by the low dislocation density and the orientation of the hard-to-deform phase structure. In contrast, the grains in the additively manufactured parts exhibit significant fragmentation, and the increased degree of recrystallization further enhances the constraints between regions, leading to a notable increase in dislocation density, as shown in Figure 10c,f. Consequently, the mechanical properties of the additively manufactured parts are determined by the high dislocation density and the presence of small-sized grains or phase structures.

## 4. Conclusions

(1) The rapid solidification in LMD components resulted in grain refinement, enhanced phase structure constraints, and a high dislocation density. Low-temperature heat treatment (700 °C and below) primarily released the high-density dislocations within the components and correspondingly reduced the yield strength.
(2) Under heat treatment at 900 °C, a significant number of recrystallized grains was promoted, leading to a further reduction in dislocation density. However, the spatial constraints between the recrystallized grains and surrounding grains generated new dislocations, resulting in a relatively small overall decrease in dislocation density in the components.
(3) High-temperature heat treatment (1100 °C) induced a transformation in the orientation of phase structures on one hand, and on the other hand, the intensification of the recrystallization process promoted the fragmentation of the dual-phase structure. As a result, the two phases no longer satisfied the optimal coordination relationship, which hinders deformation.

## Figures and Tables

**Table 1:** Lamellar eutectic dimensions of the AlFeCoCrNi_2.1_ EHEAs before and after heat treatment (μm).

		RT	500 °C	700 °C	900 °C	1100 °C
Casting	FCC	1.68	1.59	1.62	1.51	1.59
	BCC	0.66	0.58	0.62	0.62	0.62
LMD	FCC	0.62	0.63	0.63	0.61	0.74
	BCC	0.19	0.18	0.20	0.26	0.36

## Data Availability

The original contributions presented in this study are included in the article. Further inquiries can be directed to the corresponding authors.
